# A Progressive Spontaneous Cervical Compression Fracture Over Years Following Long-Term Corticosteroid Use

**DOI:** 10.7759/cureus.44628

**Published:** 2023-09-04

**Authors:** Yoshinori Maki, Motohiro Takayama, Toshinari Kawasaki, Akinori Miyakoshi

**Affiliations:** 1 Neurosurgery, Hikone Chuo Hospital, Hikone, JPN; 2 Rehabilitation, Hikari Hospital, Otsu, JPN; 3 Neurosurgery, Japanese Red Cross Otsu Hospital, Otsu, JPN; 4 Neurosurgery, Shizuoka General Hospital, Shizuoka, JPN

**Keywords:** natural course, osteoporosi, risk corticosteroid, anterior fixation, posterior fixation, cervical, vertebral compression fracture

## Abstract

Spontaneous vertebral compression fractures in the cervical region can have a significant impact on a patient's condition even after surgical management. Due to the rarity of spontaneous cervical vertebral compression fractures and the lack of a comprehensive description of this condition, the establishment of a clear understanding of its natural course remains incomplete. In this case study, a 73-year-old woman on long-term corticosteroid therapy underwent combined anterior and posterior fixation for a spontaneous vertebral compression fracture at the C3-C4 level. The vertebral compression fracture gradually worsened over a span of four years. Following the surgery, the patient experienced a temporary improvement in her neurological symptoms. However, seven months after the second operation, an instrumentation failure resulted in the patient becoming bedridden. This highlights the importance of considering the potential long-term implications and monitoring patients closely even after surgical intervention.

## Introduction

Spontaneous vertebral compression fractures can occur as a secondary result of osteoporosis, and the number of patients with this condition is increasing, especially within an aging society [1,2]. These spontaneous vertebral compression fractures are typically found in the thoracic and lumbar regions; however, only a limited number of cases involving spontaneous vertebral compression fractures in the cervical region have been described [1].

Spontaneous cervical vertebral compression fractures (SCVCFs) represent a rare occurrence that can arise from long-term corticosteroid administration, malignancy, genetic collagen disorders, and other uncommon underlying diseases [3-7]. In the management of SCVCFs, both non-operative treatments and invasive approaches such as vertebroplasty and fixation surgery have been documented [3-6]. Due to the rarity of SCVCFs, there appears to be a lack of reports detailing the natural progression of this condition.

We present a case involving an SCVCF at the C3-C4 level, resulting in the patient becoming bedridden after a two-stage combined anterior and posterior fixation procedure. We emphasize the potential for SCVCFs related to long-term corticosteroid use to worsen over an extended duration.

## Case presentation

**Figure 1:**
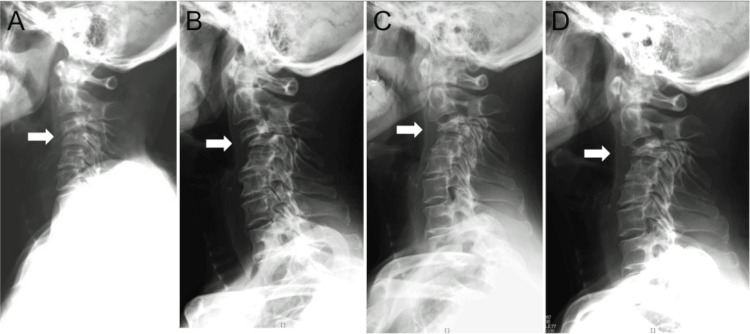
Time Course of Spontaneous Cervical Compression Fracture Progression The spontaneous cervical compression fracture exhibits progression over the span of three years, accompanied by the emergence of kyphotic changes (A: Initial X-ray image, B, C, and D: Images captured 5 months, 2 years, and 3 years after the initial visit, respectively).

A 70-year-old woman, who had been a long-term corticosteroid user due to Cronkhite-Canada syndrome, was referred to our outpatient clinic. She had not experienced any traumatic incidents. Approximately two months earlier, she had started complaining of stiffness in her neck and shoulders and had sought chiropractic treatment. However, her symptoms did not improve, and she began experiencing neck pain. An X-ray revealed an SCVCF at the C3 and C4 levels (Figure 1A). While her symptoms were not severe, the patient declined surgical intervention and opted for observational follow-up. Due to the suspicion of osteoporosis resulting from prolonged corticosteroid use, a weekly dosage of 35mg alendronate sodium hydrate was prescribed. Despite this treatment, the SCVCF worsened over the course of three years, accompanied by the progression of cervical kyphosis (Figures 1B-1D). Although surgical treatment was proposed for the progressive SCVCF, the patient did not wish for invasive treatment.

**Figure 2:**
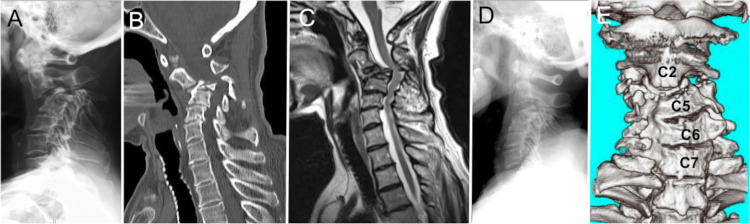
Radiological Images Upon Admission Kyphotic alterations due to the compression fracture of C3-C4 are evident. Tortuous modifications are noted in the spinal cord, although there is no apparent hyperintensity area within the spinal cord (A) X-ray image, (B) Sagittal CT image, and (C) Sagittal MR image. The kyphosis experiences slight correction with the application of a Halo-Vest (D). The midline cervical alignment continues to exhibit irregularity. (E) Three-dimensional reconstructed CT coronal image.

Three years and six months after her initial visit to our hospital (at the age of 73), the patient developed motor weakness in her upper extremities and experienced a gradual loss of hand dexterity. Eight months later, she was admitted after suddenly experiencing difficulty breathing, motor weakness in her extremities, and numbness in her lower extremities. The SCVCF at the C3-C4 level had worsened, leading to circumferential compression of the spinal cord (Figures 2A-2C). In response, the patient's neck was promptly immobilized using a cervical collar. The patient did not agree with open surgery even after this event. Subsequently, her neck was manually repositioned and stabilized using a Hallo-vest (Figures 2D, 2E).

To safeguard the spinal cord, corticosteroid treatment was administered. This approach alleviated the dyspnea, and her other symptoms did not worsen.

**Figure 3:**
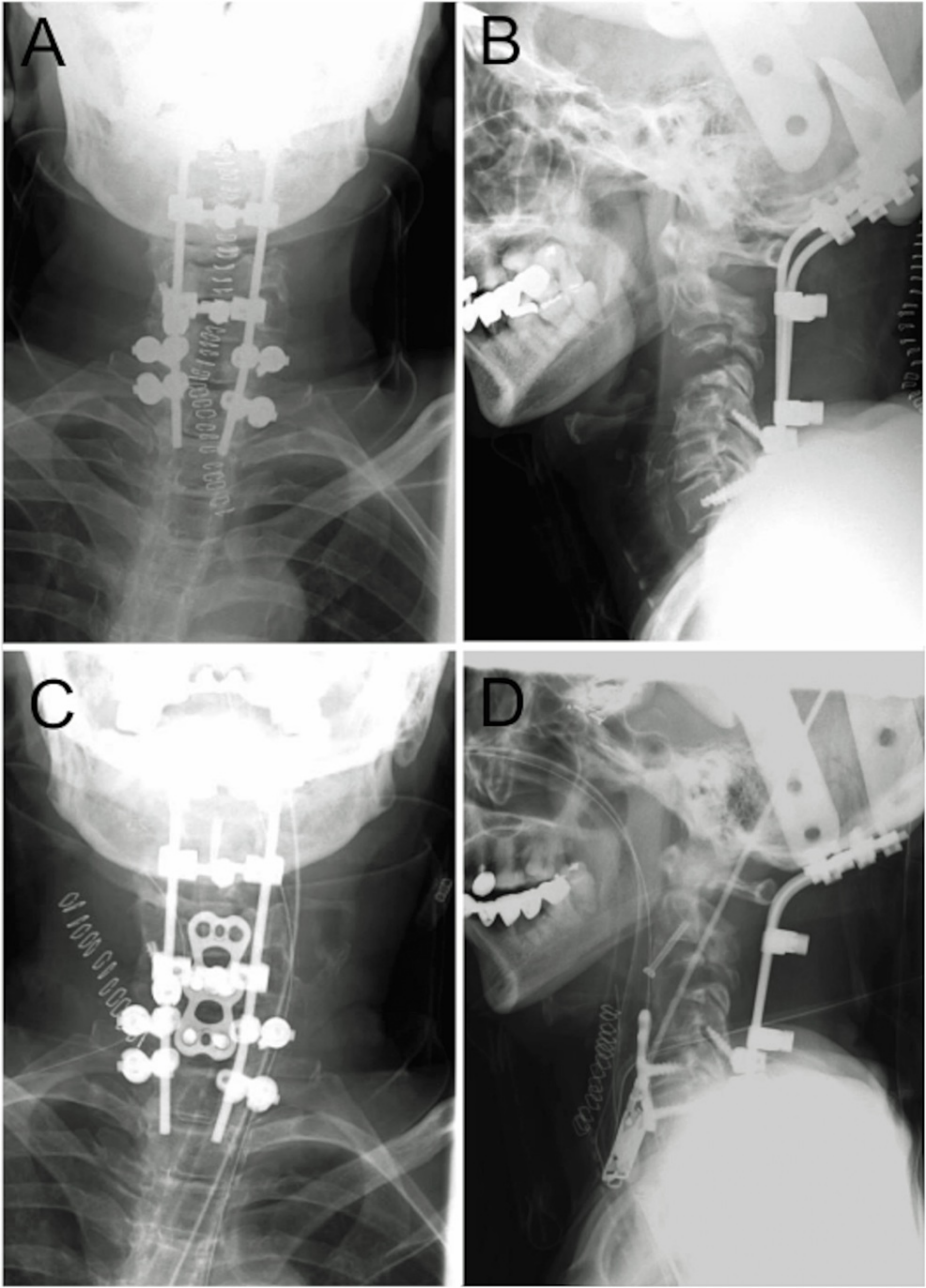
X-ray Images Post-First and Second Operations Cervical alignment achieves correction through posterior fixation extending from the occipital bone to Th1 (A, B). Anterior cervical alignment is supported through anterior fixation utilizing a canulated mini screw to secure C2 and a fibula graft, in addition to a titanium plate for fixation of the graft bone and the vertebral bodies of C5 and C6 (C, D).

Three weeks after the onset of symptoms, as we obtained informed consent from the patient, we planned combined anterior and posterior fixation surgery. We thought that circumferential surgery in a single session could be quite invasive for the patient. Thus, we performed the operation in two sessions. Posterior decompression and fixation surgery was preceded to protect the spinal cord because we thought preceding anterior approach could lead to spinal cord injury intraoperatively. A laminectomy was performed from C2 to C6, followed by posterior fixation extending from the occipital bone to Th1 (Figures 3A, 3B). Because the patient was a corticosteroid user, a skin incision small enough seemed warranted to avoid postoperative wound complications. In addition, we though that the fixation level of Th1 would be caudal enough from the SCVCF. A week after the first procedure, an anterior approach surgery was conducted. The vertebral bodies of C3 and C4 were replaced with a fibula graft. C2 and the grafting material were secured using canulated mini screws. The fibula graft and the vertebral bodies of C5 and C6 were stabilized with a titanium plate fixation (Figures 3C, 3D).

**Figure 4:**
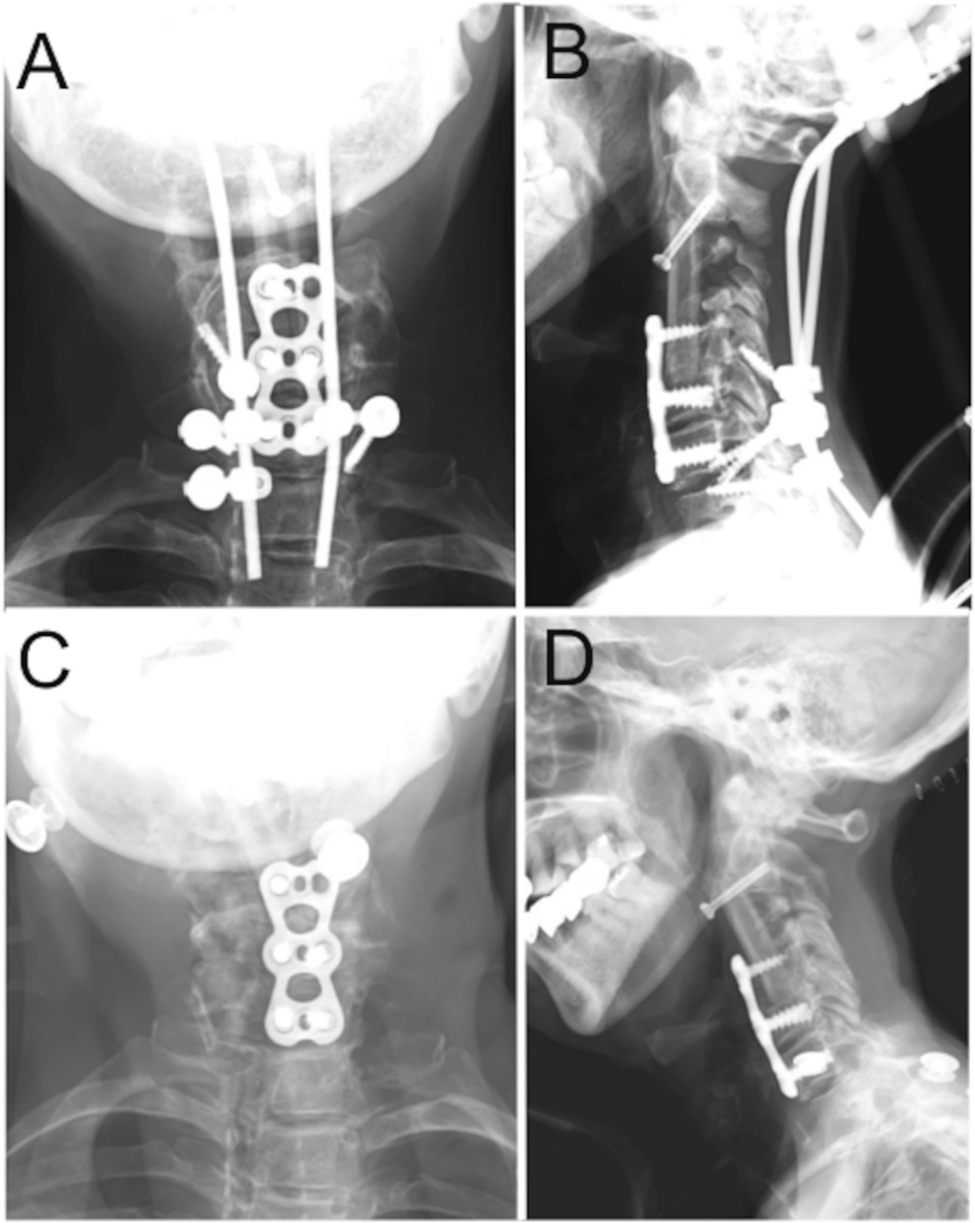
X-ray Images Following Instrumentation Failure Due to instability, the left Th1 pedicle screw is removed (A, B). Fusion does not occur between the fibula graft and C2 vertebral body, leading to the displacement of the canulated mini screw (C, D).

Following the surgery and subsequent rehabilitation therapy, the patient was discharged from our hospital. However, four months after discharge, she returned with complaints of pain in her left upper extremity. The pedicle screw on the left side of Th1 had shifted backward, necessitating surgical removal (Figures 4A, 4B). This pedicle screw instability was followed by a complete failure of posterior stabilization. Consequently, all pedicle screws and rods were removed. This complication resulted in the destabilization of the graft placed during the anterior approach (Figures 4C, 4D).

As a consequence, the patient experienced dyspnea when sitting but declined further surgical intervention. Ultimately, she became bedridden and was transferred to a long-term care facility.

## Discussion

We present a case of SCVCF of C3-C4 that progressed over an extended period, leading to combined anterior and posterior cervical fixation. However, possibly due to the underlying osteoporosis related to long-term corticosteroid use, the instrumentation did not provide stabilization. Consequently, the patient's ability to carry out daily activities deteriorated, resulting in a bedridden status.

Spontaneous VCFs are typically observed in the thoracic and lumbar spine, with reports of this condition in the cervical spine being rare [1]. Underlying conditions potentially causing SCVCFs have been reported, such as osteogenesis imperfecta, Gorham-Stout disease, breast cancer metastasis, and long-term corticosteroid administration [3-6,8]. Although the vertebral bodies of C3 and C4 were not examined pathologically in our case, long-term corticosteroid administration is likely the most significant risk factor for spontaneous VCFs.

Given the rarity of cervical VCFs, standardized surgical management remains uncertain. In the study by Fisahn et al., a spontaneous cervical fracture from C4 to C6 resulted in anterior fixation of C4-C6 and posterior fixation of C2-T1, with good postoperative recovery after nine weeks. This favorable outcome could be attributed to the patient's relatively young age (41 years old), contrasting with our patient's age [3]. In cases like a C4 compression fracture due to Gorham-Stout disease, treatment involved anterior fixation of C3-C5 and posterior fixation of C3-C5 (extended to T4 in an additional surgery) [4]. Similarly, a C6 compression fracture due to osteogenesis imperfecta was addressed with anterior fixation of C5-C7 and posterior fixation of C4-C7 [5]. Vertebroplasty was performed for a C3 compression fracture due to breast cancer metastasis [6]. A spontaneous compression fracture of C7 due to leiomyosarcoma only underwent a biopsy [7] (Table 1).

**Table 1:** Previous Reports of Spontaneous Cervical Compression Fracture

	Sex, age (years)	Underlying disease	Level of spontaneous vertebral compression fracture	Treatment
Fisahn et al., 2016 [3].	Woman, 41	Long-term corticosteroid administration	C4 - C6	Anterior fixation of C4-C6 and posterior fixation of C2-T1
Kim et al., 2019 [4].	Man, 22	Gorham-Stout disease	C4	Anterior fixation of C3-C5 and posterior fixation of C3-C5 (extended to T4 in an additional surgery)
Leng et al., 2010 [5].	Woman, 15	Osteogenesis imperfecta	C6	Anterior fixation of C5-C7 and posterior fixation of C4-C7
López-O’Rourke et al., 2009 [6].	Man, 43	Breast cancer metastasis	C3	Vertebroplasty
Ochiai et al., 2000 [7].	Man, 69	Leiomyosarcom	C7	Biopsy

The optimal timing for surgical intervention in cases of SCVCFs is also a subject of debate. Initial symptoms of this condition might not be severe, as was the case in our patient's initial visit to our institute. However, considering that SCVCFs can worsen over time, as observed in our case, early surgical intervention might be advisable. Additionally, our patient did not initially receive medication for osteoporosis following corticosteroid use, and instrumentation failure occurred despite the subsequent initiation of alendronate sodium hydrate. Early screening and identification of long-term corticosteroid patients who are not receiving osteoporosis-preventing medication could contribute to better outcomes after fixation surgery for SCVCFs.

Surgical strategy for cases with SCVCFs similar to our case should also be discussed. As osteoporosis is considered to be a risk for revision surgery in cervical regions [9], strong fixation seems desirable. As instrumentation failure occurred from the Th1 pedicle screw in our case, the caudal level in posterior fixation surgery might not have been long enough. A case with osteoporosis with an underlying disease for which posterior fixation surgery of occiput to Th3 was described [10]. In the previous case, posterior fixation surgery was performed for dens fracture and bilateral C2 facet fracture resulting from ankylosing spondylitis [10]. The fracture level and underlying disease in the previous case were different from those in our case. Therefore, it remains questionable whether the same surgical strategy can be applied to cases similar to ours. 

Posterior fixation from the occipital bone to the middle or lower thoracic levels more caudal to the kyphotic apex can result in stabilizing instrumentation. However, due to the administration of corticosteroid in our case, we judged that posterior long fusion with a ling skin incision could have led to postoperative wound complications. Therefore, additional anchors such as replacement of pedicle screws of C2 could have been useful to avoid the instrumentation failure. 

## Conclusions

An SCVCF can develop in patients with long-term corticosteroid use. This rare condition can worsen over the years, leading to combined anterior and posterior cervical fixation. In our case, the failure of instrumental stabilization resulted in the patient becoming bedridden. This complication may have arisen due to pre-existing untreated osteoporosis linked to long-term corticosteroid use. Clinicians should exercise caution, as SCVCFs can exhibit prolonged progression and have serious consequences. Thus, the optimal timing and strategy for surgical intervention should be thoroughly assessed.
